# Evaluation of the Strength of the Interface for Abaca Fiber Reinforced Hdpe and Biope Composite Materials, and Its Influence over Tensile Properties

**DOI:** 10.3390/polym14245412

**Published:** 2022-12-10

**Authors:** Faust Seculi, Francesc X. Espinach, Fernando Julián, Marc Delgado-Aguilar, Pere Mutjé, Quim Tarrés

**Affiliations:** LEPAMAP-PRODIS Research Group, University of Girona, 17003 Girona, Spain

**Keywords:** biopolymers, natural fibers, micromechanics, interface, green composites

## Abstract

In this study, tensile properties of abaca-reinforced HDPE and BioPE composites have been researched. The strength of the interface between the matrix and the reinforcement of a composite material noticeably impacts its mechanical properties. Thus, the strength of the interface between the reinforcements and the matrices has been studied using micromechanics models. Natural fibers are hydrophilic and the matrices are hydrophobic, resulting in weak interfaces. In the study, a coupling agent based on polyethylene functionalised with maleic acid was used, to increase the strength of the interface. The results show that 8 wt% coupling agent contents noticeably increased the tensile strength of the composites and the interface. Tensile properties obtained for HDPE and BioPE-based coupled composites were statistically similar or better for BioPE-based materials. The use of bio-based matrices increases the possibility of decreasing the environmental impact of the materials, obtaining fully bio-based composites. The article shows the ability of fully bio-based composites to replace others using oil-based matrices.

## 1. Introduction

Composite materials have the advantage of combining the properties of their phases to obtain new materials with enhanced properties. These properties can be related to increasing mechanical performance or decreasing costs and environmental impacts, among other factors. Ideally, any material that shows positive performances in all the mentioned aspects or acquires novel applications will acquire sound competitive advantages [1,2,3,4]. The literature shows different approaches to obtaining composite materials with these desired advantages [5,6,7,8]. The industry point of view is based on obtaining greener materials with mechanical properties equal to commercial products, which allow for adaptation to present and future regulatory frameworks as well as maintaining or reducing costs [5]. To increase the mechanical properties of bio-based matrices, the use of plasticisers, reinforcements, or Nano reinforcements has been explored [7]. Another approach is the modification of the fiber surfaces to increase their compatibility with the matrices, by fiber treatment with the use of coupling agents [7,8].

In the case of composite materials where the matrix is a polymer, increasing the mechanical performance usually involves the use of reinforcements in the shape of long or short fibers. Short fibers are used for materials to be transformed by injection or extrusion molding, processes commonly used by the industry [9]. Mould-injected or extruded materials, reinforced with short fibers, increase their stiffness measured using Young’s moduli, as a consequence of adding a stiffer phase to the composite. When the interface between the fiber and the reinforcement is strong, they also increase the tensile strength of the matrix [10,11,12,13]. Moreover, due to the rigidity of the reinforcements, composite materials tend to show more fragile breaking mechanisms and a lower ability to sustain deformations without breaking than the matrix. Composite materials usually show a lower impact strength than ductile matrices [14,15,16]. Glass fiber (GF) is the reinforcement most used by the industry, and its composites can be considered commodities. GF, as a man-made reinforcement, offers high reliability and regularity in its properties, is a cheap reinforcement, and has shown high strengthening and stiffening abilities when used with polyolefins such as polypropylene (PP) or high-density polyethylene (HDPE) [16,17,18,19]. Nonetheless, GF show some drawbacks. GF-reinforced composites are difficult to recycle as the fibers are fragile and tend to shorten from one cycle to the other, decreasing their strengthening and stiffening abilities [20,21]. Moreover, GF is harmful to the operators and has been related to dermatitis and breathing problems [22,23,24]. Moreover, GF is abrasive and reduces the useful cycles of machinery and accessories. Hence, the use of mineral fibers as reinforcement is a good means to obtain composite materials with increased mechanical properties but cannot be seen as the way to obtain cheaper or more environmentally friendly materials.

Some efforts in the direction of obtaining materials more environmentally friendly than GF-reinforced composites have been made by using natural fibers instead of mineral ones [25,26]. Natural fibers come from a renewable source and in some cases can be obtained from agroforestry waste in the shape of straws, stalks, or stems. Thus, their use allows for the enlargement of the value chain of agroforestry and at the same time reduces possible incinerations of such byproducts in the fields. Moreover, natural fibers are less abrasive than GF and prevent machinery wear. At the same time, natural fibers are less harmful to users than GF. Notwithstanding, natural fibers tend to show less regularity in their mechanical properties and morphology than GF. Another drawback of natural fibers as polyolefin reinforcement is their polarity. Polyolefins are hydrophobic, and the surface of natural fibers is hydrophilic, complicating the possibility of obtaining strong interfaces. To solve the incompatibilities between the phases of the matrix, the literature shows the benefits of using some natural fiber treatments such as silane treatment or the use of coupling agents [27,28,29]. Some authors emphasise that the use of natural fibers serves the three goals at the same time. On the one hand, the literature shows that obtaining composite materials with competitive mechanical properties by using natural fibers as reinforcement is possible [25,26,29,30,31]. On the other hand, when one of the phases of the composite has a cost noticeably lower than the other phase, the resulting material is cheaper than the original matrix. Thus, even when natural fibers act as fillers and do not increase the mechanical properties of the composite, they can reduce the final cost of the material. In addition, taking into account that natural fibers come from renewable sources, a priori, their composites seem to be more sustainable. There is some literature in this direction [32,33,34,35]. Nonetheless, some researchers argue that to establish the sustainability of a material it is mandatory to make a life cycle analysis (LCA) [36,37,38]. Thus, if a strong interface between the phases is obtained, natural fibers can be used to obtain competitive materials with increased mechanical properties which are cheaper than GF-reinforced composites. Theoretically, natural fiber-reinforced materials seem more sustainable than materials reinforced with mineral fibers, but to sustain this an LCA must be performed.

Bio-based plastics were developed to obtain polymers from renewable sources and avoid dependence on oil. These materials are obtained from the selective transformation of plants and other non-fossil sources [39]. Thus, it is possible to obtain fully bio-based composite materials by reinforcing a bio-based plastic with natural fibers. These green composites have the potential to reach the same mechanical properties as oil-based polymers reinforced with natural fibers, and the literature shows that such materials are competitive [33,40,41,42]. Moreover, both being phases of the composite bio-based, it can be expected, a priori, that these materials will be more environmentally friendly than oil-based ones. Last but no less important is the cost of these bio-based materials. Nowadays, bio-based plastics are more expensive than oil-based ones. The literature points out that there are some scale economy issues there, and if the production of bio-based plastics increases, costs will decrease. In any case, a strategy of incorporating cheap natural fiber reinforcements into bio-based plastics can be used to equalise the costs of such matrices [43].

Abaca is a bast fiber extracted from the stalk of the plant. The abaca is also known as Manila hemp. It is a Musasea family plant native to Asia and is planted in humid areas including in the Philippines and the east of Indonesia. Abaca fibers are used extensively to produce ropes, woven fabrics, tea bags, etc. It is also called a biodegradable and sustainable fiber. Abaca is considered the strongest of natural fibers, is more resistant to saltwater decomposition than most vegetable fibers, and possesses higher tensile strength and lower elongation in both wet and dry states in comparison to rayon and nylon fiber [44]. The Philippines is the world’s largest producer and supplier of abaca fiber for cordage and pulp for specialist paper. It supplies 85% of the needed abaca fiber around the globe.

The authors’ research is based on the replacement of oil-based matrices and man-made reinforcements with bio-based materials to obtain sustainable materials with properties similar to commercial materials based on nonrenewable sources. To the best knowledge of the authors, the literature shows multiple papers on the usage of natural fibers as polyolefin reinforcement, but few explore the use of natural fiber as a reinforcement of bio-based matrices. In the case of abaca fibers and BioPE, the authors have not found any reference. In this paper, composite materials based on HDPE and BioPE matrices with similar tensile properties were used to obtain composite materials reinforced with 30 wt% of abaca fibers. A coupling agent was added to the materials and was used to evaluate its effect on the tensile properties of the composites. Abaca fibers were chemically characterised to understand their potential for bonds created with the coupling agent. Furthermore, a single fiber test analysis was used to discover the intrinsic tensile strength of the fibers. To ensure strong interfaces, abaca fibers were silane treated and percentages ranging from 0 to 10 wt% of polyethylene functionalised with maleic acid (MAPE) were added to composites containing 30 wt% of AF. The composites showing the highest tensile strengths were identified as the ones with the strongest interfaces. The materials were tensile tested to obtain their tensile strength, Young’s modulus, and strain at rupture. The differences and similarities between oil-based and bio-based composites were discussed and compared with commodity materials used by automotive, construction, and product design industries, such as glass fiber reinforced polyolefin. To evaluate the contribution of the phases and the strength of the interface, micromechanics analyses of the tensile strength and Young’s modulus were performed. These analyses allowed the evaluations of the potential of the composite materials and also the potential of the fiber-matrix interface to be performed. The paper shows the ability of BioPE to replace HDPE for natural fiber-reinforced composites. BioPE- based composites show properties in line with or better than their HDPE-based counterparts. The strength of the interphase between BioPE-based composites and natural fibers increases when MAPE is added to the formulation of the materials, and is in line with their HDPE counterparts. The research shows that, while the strength of the interfaces can be cataloged as strong, micromechanics models such as a modified rule of mixtures predict that such interface strength can be increased. BioPE-based materials showed higher strains at break than HDPE-based materials.

## 2. Materials and Methods

### 2.1. Materials

A high-density polyethylene (HDPE) and a bio-based high-density polyethylene (BioPE) with references HDI0661U1by and SHA7260, respectively, were used as matrices. Both polymers were kindly supplied by Braskem (Sao Paulo, Brazil). To improve the compatibility between the matrices and the reinforcements, a coupling agent consisting of polyethylene functionalised with maleic acid (MAPE) was added to the formulations of the composite materials. This coupling agent was acquired from DuPont (Wilmington, DE, USA) with commercial reference Fusabond^®^ MB100D.

Filipino abaca strands, provided by CELESA (Tortosa, Spain), were used as natural fiber reinforcement.

### 2.2. Abaca Fiber Modification and Treatment

The surface of the abaca strands was treated with hexadecyltrimethoxysilane. This reactant, with reference Dynasilan^®^ 9116 from Degussa Iberia SA (Barcelona, Spain), was used to block the hydroxyl groups on the fiber surface. The silane was hydrolised at 40 °C, PH4 for 2 h at 2 wt% solution in 1-methoxy-2-propanol. Abaca strands were added to the solution and continuously stirred for 2 h, and then filtered and dried at 80 °C for 24 h.

### 2.3. Chemical Characterisation of the Fibers

Raw abaca fibers were dried in an oven at 105 °C until they reached constant weight. Then, the dried fibers were milled and screened as described in TAPPI standard T257. The solvent extractives determination was made according to TAPPI standard T204. Then, 5 g of material was submitted to Soxhlet extraction for 5 h in the presence of 150 mL of ethanol-toluene. Afterwards, the extract was dried until it reached constant weight.

Klason lignin was determined according to TAPPI standard T222. In this case, 80 mL of cold sulfuric acid at 72% was gradually added to 4 g of the sample. Then, the beaker was kept in a bath at 20 °C for two hours with stirring, and then transferred to a flask containing 1000 mL of deionised water which was boiled for 4 h. After this, the flask was left inclined overnight to cause lignin precipitation.

Cellulose contents were obtained by high-performance anion exchange chromatography (HPAEC). Therefore, samples were hydrolysed in sulfuric acid and diluted with deionised water. Samples and standard solutions were stored in an autoclave for 60 min at 125 °C, filtered, and washed. The filtered sample was used for chromatographic determination. Hemicellulose was quantified by the difference from 100%.

### 2.4. Single Fiber Tensile Test

Individualised fibers of abaca were tensile tested following ASTM D3822-01. The tensile tests were carried out in an INSTRON 5500R testing device (supplied by INSTRON, Cerdanyola del Vallès, Spain) equipped with a 5 kN force cell. The experiments were repeated for 4 different gauge lengths: ¼”, ½”, ¾”, and 1”, with 2.54, 1.91, 1.27, and 0.65 mm/min cross speeds, respectively. At least 100 fibers were measured for every gauge length.

### 2.5. Composites Fabrication

All the composites were compounded in an intensive melt mixer Brabender^®^ Plastograph. The process was carried out at 180 °C for 10 min and at 80 rpm. These process parameters have been used by researchers in the past and ensure good fiber dispersion while minimising reinforcement shortening [30]. Once the composites were extracted from the mixer and cooled down, they were pelletised into particles with a mean diameter of 5 mm in a hammer mill. Uncoupled composites, adding 30 wt% of Abaca fibers (AF) as reinforcement of HDPE and BioPE, were prepared and tensile tested. Then, composite materials, adding from 2 to 10 wt% of MAPE, were prepared and tensile tested. The results were used to establish the percentage of MAPE that rendered the highest tensile strength. The percentage of MAPE was calculated against AF content.

### 2.6. Standard Specimen Obtention

Before their mould injection, the pellets were stored at 80 °C for 24 h to eliminate the humidity. Then, a mould injection machine Meteor^®^ 40 by Mateu and Solé (Barcelona, Spain) was used to obtain dog bone standard specimens in agreement with ASTM D638. The machine has three heating areas that were programmed at 175 °C for the first and the second and 190 °C for the nozzle. Composites were mould injected in a steel mould at 120 kg/cm^2^ and a maintenance pressure of 25 kg/cm^2^.

### 2.7. Testing the Tensile Properties of the Composites

According to the ASTM D638, the specimens were stored in a conditioning chamber at 23 °C and 50% relative humidity for the 48 hours before their tensile testing. Specimens were placed in an Instron^®^ 1122 Universal testing machine, acquired from Metrotec, S.A (Barcelona, Spain). The machine is equipped with a 5 kN load cell and allows tensile testing of the specimens in agreement with ISO 527-1:2000. At least 5 specimens for any of the composite formulations were tensile tested, and the experimental results are the mean values of such tests. An extensometer was used to evaluate the deformations used to compute Young’s moduli.

ANOVA analyses of the results were made with R^®^ and RCommander at a 95% confidence interval.

## 3. Models Used for Tensile Strength and Young’s Modulus Micromechanics

Macro mechanics considers materials homogeneous whether they are a single material or a composite, and studies their behavior under known contour conditions. The results are of interest to researchers and practitioners as they will be used to preview the behavior of any product manufactured using the materials. On the other hand, micromechanics is interested in the behavior of composite materials under described contour conditions. In this case, the material is considered heterogeneous with distinct phases. The main concern of micromechanics is the relationship between the phases of a composite and how the properties of the phases affect the properties of the composite. The results are interesting when formulating composite materials with target properties.

Micromechanics uses experimental values to preview the mechanical properties of a composite. Thus, it is of interest to know the parameters that impact the properties of a composite. In the case of semi-aligned short fiber reinforced polymer composites, such parameters are the nature, content, and properties of the phases, the compatibility between such phases, the grade of homogeneity of the material, or the degree of dispersion of the reinforcements in the matrix, the mean orientation of the reinforcing fibers concerning the applied loads, the morphology of the reinforcements, mainly its aspect ratio (fiber length/fiber diameter), and finally, the nature and strength of the interface between the matrix and the reinforcements. Thus, any micromechanics model will take in account these parameters as the minimum. Nevertheless, the literature agrees that the strength of the interface has less impact in the case of Young’s modulus of the composites than in their tensile strength [45].

Multiple micromechanics models can be used to preview the properties of composite materials, but a high amount of such models come from a rule of mixtures. In the case of Young’s modulus of semi-aligned short fiber reinforced polymer composites, a formulation of a modified rule of mixtures is as follows.

(1) $$E_{t}^{C}=\eta_{e}\cdot E_{t}^{F}\cdot V^{F}+\left(1-V^{F}\right)\cdot E_{t}^{M}$$

where Young’s modulus of the composite, reinforcement, and matrix are represented by $E_{t}^{C}$, $E_{t}^{F}$, and $E_{t}^{M}$, respectively, and *V^F^* is the volume fraction of the reinforcement. $\eta_{e}$ is a modulus efficiency factor used to correct the stiffening efficiency of the fibers. Such a factor depends on the orientation and morphological features of the fibers.

The modified rule of mixtures was originally developed for Young’s modulus of short fiber reinforced composites (cites). Nonetheless, such an equation can be easily adapted for the tensile strength of such composites, as follows:

(2) $$\sigma_{t}^{C}=f_{c}\cdot \sigma_{t}^{F}\cdot V^{F}+\left(1-V^{F}\right)\cdot \sigma_{t}^{m*}$$

where *σ_t_^C^* and *σ_t_^F^* are the tensile strength of the composite and the intrinsic tensile strength of the reinforcement, respectively, and σ_t_^m*^ is the contribution of the matrix at the ultimate strain of the composite. To account for the strength of the interface, the mean orientation of the reinforcements and its morphology, the contribution of the fibers is multiplied by a coupling factor (*f_c_*). It is accepted that well-bonded composites show coupling factors ranging from 0.18 to 0.2 [46,47].

Both modified rules of mixtures present two unknowns, and as such cannot be solved. In the case of Young’s modulus, these are the intrinsic Young’s modulus of the reinforcements and the modulus efficiency factor. In the case of the tensile strength, the unknowns are the intrinsic tensile strength of the fibers and the coupling factor. These mechanical properties are unknown and difficult to measure experimentally because natural fibers show a high scatter in their mechanical properties and morphology when measured individually. Moreover, some authors prefer using micromechanical methods to evaluate such properties [48].

In any case, $\eta_{e}\cdot E_{t}^{F}$ in Equation (1) and $f_{c}\cdot \sigma_{t}^{F}$ in Equation (2) account for the net contribution of the reinforcements to Young’s modulus and the tensile strength of the composite, respectively. Consequently, Equations (1) and (2) can be rewritten by isolating the abovementioned contributions. Then, a Fiber Tensile Modulus Factor (FTMF) and a Fiber Tensile Strength Factor (FTSF) can be defined. These factors, which are the slope of the regression curves of the contributions of the fibers at different contents, are a measure of the potential stiffening and strengthening capabilities of any reinforcement and have been used previously in the literature [30,31,45].

The FTMF will be computed from the following Equation:

(3) $$\eta_{e}\cdot E_{t}^{F}=\frac{E_{t}^{C}-E_{t}^{M}\cdot \left(1-V^{F}\right)}{V^{F}}$$

The FTMF is obtained from the slope of the line by representing $E_{t}^{C}-E_{t}^{M}\cdot \left(1-V^{F}\right)$ against $V^{F}$ at each fiber content.

Likewise, the FTSF can be obtained using:

(4) $$f_{c}\cdot \sigma_{t}^{F}=\frac{\sigma_{t}^{C}-\left(1-V^{F}\right)\cdot \sigma_{t}^{m*}\;}{V^{F}}$$

As mentioned, the intrinsic Young’s modulus and strength of the fibers can be obtained experimentally by single fiber tests or by nanoindentation [48,49,50]. However, natural fibers tend to show high scatter due to their nature, and some researchers prefer using additional micromechanics models to obtain some of the intrinsic values. In the case of the intrinsic Young’s modulus, Hirsch equations can be used for this purpose.

The Hirsh equation is a linear combination of Reuss and Voigt models [51]:

(5) $$E_{t}^{C}=\beta \cdot (E_{t}^{F}\cdot V^{F}+E_{t}^{M}\left(1-V^{F}\right)+\left(1-\beta \right)\frac{E_{t}^{F}\cdot E_{t}^{M}}{E_{t}^{F}\cdot V^{F}+E_{t}^{M}\left(1-V^{F}\right)}$$

where *β* is a parameter that equalised the contributions of fibers theoretically aligned or perpendicular to the loads. In the case of semi-aligned short fibers, reinforced composites’ *β* has a value of 0.4 [52].

## 4. Results

### 4.1. Chemical Composition of Abaca Fibers

Table 1 shows the obtained chemical composition of Abaca fibers used to obtain the composite materials, as well as the chemical composition of other fibers obtained from the literature [44,53].

The fibers represented in the table are the ones with higher cellulose and hemicellulose contents. Cellulose is considered a natural polymer and is the main structural constituent of natural fibers. Its content is positively correlated with the intrinsic mechanical properties of the fibers [53]. Thus, fibers with high cellulose contents are expected to be stronger and stiffer than those with lower cellulose contents. Abaca fibers used in the research showed a high content of cellulose, comparable to the other fibers. Fibers presented in Table 1 are non-wood plants that present lesser contents of lignin and higher cellulose contents than wood fibers [45]. On the other hand, cellulose has a negative correlation with the strain at failure. This is a consequence of the lower percentages of lignin, responsible for the structural maintenance of the cell wall. Thus, these natural fibers with high cellulose contents are expected to show a brittle behavior, with high tensile strengths and Young’s moduli, but, compared with wood fibers, lower strains at the break. The fiber with higher cellulose contents is cotton, but this means a very low lignin content that hinders its dispersion during mixing operations [54]. Cellulose contents are also needed to ensure the proper exploitation of the coupling agent’s ability to create bonds between the maleic acid and the hydroxyl groups of the cellulose.

Hemicelluloses create short branching chains that embed cellulose in a matrix. Their presence is positively correlated with the intrinsic Young’s modulus and tensile strength of the fibers. Abaca fibers show percentages of hemicellulose similar to the other natural fibers listed in Table 1, except for cotton, which shows noticeably lesser amounts.

Lignin is the chemical adhesive between fibers. Lignin, as commented above, has a positive correlation with the strain at failure and a negative correlation with the tensile and Young’s modulus of the fibers. Thus, low percentages of lignin, such as the ones shown by abaca fibers, grant strong and stiff fibers.

Due to the chemical composition of the abaca fibers used in the research, if a strong interface is obtained, the composites must show noticeable increases in their tensile properties. On the other hand, a noticeable decrease in the ability of the materials to deform without breaking is also expected.

### 4.2. Mechanical Characterisation of Abaca Fibers

Table 2 shows the mean experimental intrinsic tensile strengths (*σ_t_^F^*) obtained from the single fiber tensile tests. The gauge lengths of the equipment used to tensile test the fibers were established at four different positions; 1, ¾, ½, and ¼ inches. A minimum of 100 fibers were tested for each gauge length. The strength of a fiber is negatively correlated to its length because the probability of finding a defect increases with the length of the fiber. Moreover, due to their nature, natural fibers show high standard deviations in their mechanical properties. The experimental values showed the expected correlation between fiber length and intrinsic strength.

The experimental results obtained from the single fiber tests of abaca fibers were used to evaluate the probability of their failure under stress, using a Weibull analysis. This probability under given stress is related to the presence of critical defects in the fiber surface that allow crack propagation [55]. The failure stress probability can be described according to a Weibull distribution:

(6) $$P_{f}\left(\sigma \right)=1-e^{\left[-{\left(\frac{\sigma }{\eta }\right)}^{\beta }\right]}$$

In the equation, *β* is the Weibull modulus and evaluates the scatter of the strength values. The higher the Weibull modulus is, the shorter the dispersion of the strength values. In the equation, *σ* and *η* are the experimental intrinsic tensile strengths and the characteristic strength of the fiber, respectively.

**Figure 1:**
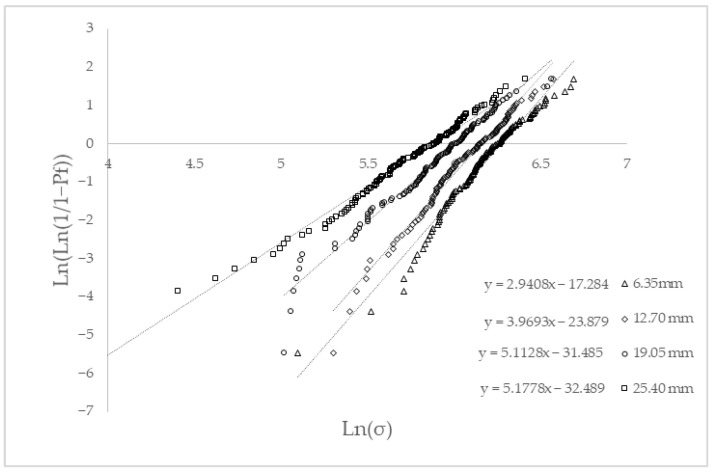
Linearised cumulative probability of failure against the natural logarithm of the measured abaca fiber tensile strengths for each gauge length.

Figure 1 shows the linearised cumulative representation of the failure probability versus the natural logarithm of the obtained intrinsic fiber tensile strengths. The figure shows the results obtained for four gauge lengths.

Weibull shape factors showed low values, below 10, denoting the obtained wide scatter of the single fiber intrinsic tensile strengths. The mean intrinsic tensile strengths are similar but lower than the respective characteristic strengths. The characteristic strengths show an increasing probability of failure when the length of the fibers increases. The characteristic strength of the fibers evolved linearly with the fiber length (*L^F^*) between ¼” and 1”, accordingly to the following equation:

(7) $$\sigma_{t}^{F}=589-9.2126\cdot L^{F}$$

If the fibers are extracted from a composite, they show a fiber length distribution with lengths mainly below 1 mm. Equation (7) can be used to obtain an upper bound for a theoretical maximum intrinsic tensile strength of the fibers: 589 MPa when its length is 0. This intrinsic tensile strength can be used to model the properties of the composites and obtain upper bounds for their tensile strength, or to characterise a lower bound for the strength of the interface. The literature reports the intrinsic tensile strength of abaca fiber ranging from 400 to 1135 MPa [45,56,57,58]. The measured values are inside this range.

### 4.3. Tensile Strength of the Composites

Modified rules of mixtures (Equations (1) and (2)) show the effect of different parameters over the contributions of the phases to the mechanical properties of the composites. In the case of the tensile strength, this property is impacted by the contents of the phases, a proper dispersion of the reinforcement on the matrix, the morphology of the fibers, the mean orientation angle of such fibers, and the strength of the interface between the fibers and the matrix. In the case of Young’s modulus, the parameters are the same except for the strength of the interface [59].

Untreated or non-modified natural fiber reinforced polyolefin composites show weak interfaces [32,60]. This is due to the different polarities of the phases. On the one hand, natural fibers are hydrophilic, and on the other hand, the polyolefin is hydrophobic, thus, the interface is limited to the mechanical anchorage between the phases. Moreover, interfaces of untreated composites tend to show separations and voids in the interface region. To reinforce the interface, the literature shows different strategies [30,61]. The interface is the contact zone between the phases of the composite. There, the phases can be combined mechanically, chemically, or physically, resulting in strong or weak interfaces depending on the amount or strength of the cited combinations. To increase chemical bonds between the fiber surface and the matrix, this surface can be modified to reduce its hydrophobicity. The treatment includes solvent extraction, plasma treatments, heat treatments, γ-ray, UV bombardment, and chemical modifications. A common solution is the use of maleic acid maleated polymers [10]. This coupling agent avoids using other expensive and toxic reagents to obtain similar results. The coupling agent interacts, on the one hand, entangling with the polymer chains of the matrix through interdiffusion phenomena. On the other hand, it creates covalent bonds with the hydroxyl groups in the fiber surfaces.

Table 3 shows the effect of 0 to 10 wt% MAPE contents on the tensile properties of composites reinforced with 30 wt% of AF.

**Figure 2:**
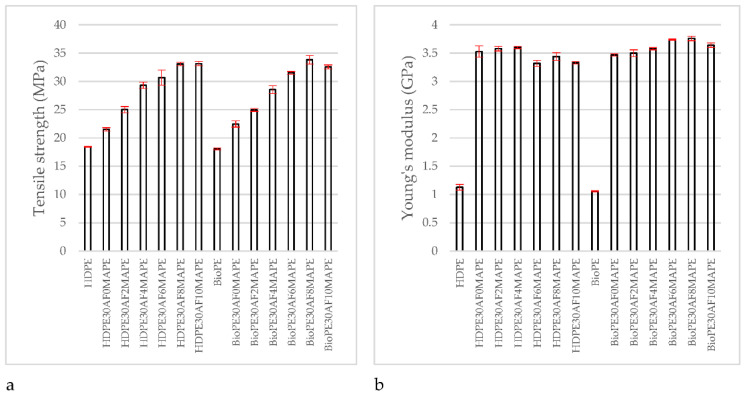
Evolution of the tensile properties of Abaca fiber reinforced HDPE composites against MAPE content: (**a**) Tensile strength; (**b**) Young’s modulus.

Adding coupling agents to composite materials noticeably increased their tensile strength and their Young’s modulus, despite the matrix. It was found that the tensile strength increased with the amount of coupling agent, but Young’s modulus returned more regular values at any coupling agent content. Figure 2 shows the different behaviors of tensile strength and Young’s modulus against coupling agent contents.

Table 3 shows that the tensile strength, Young’s modulus, and strain at break of all the composite materials can be considered statistically different from the values of the matrix. This was expected, especially in the case of Young’s modulus, because adding a more stiff and fragile phase to a composite will increase its stiffness, but will also affect its ability to deform without breaking, thus decreasing its strain at break. The tensile strength of composite materials is heavily impacted by the strength of the interface, and if the interface is weak, its tensile strength can be lower than the matrix [24].

Tensile strength increased noticeably for MAPE contents in the range from 2 to 8 wt%. Then, in the case of HDPE, composites with a 10% coupling agent content showed a slight but not statistically significant increase. In the case of BioPE, tensile strength decreased slightly. On the other hand, the differences between the tensile strength of the composites adding 6 or 8 wt% of MAPE were statistically significant. Thus, the experiment showed that an 8 wt% MAPE content delivered the highest tensile strengths for the composite materials reinforced with 30% of abaca fibers. Moreover, the tensile strengths obtained for the HDPE and the BioPE-based composites did not show statistically significant differences. This MAPE content did not deliver the highest Young’s modulus in the case of HDPE-based composites, which were achieved with a 4 wt% of MAPE. Table 3 shows that Young’s moduli obtained with 4 and 8 wt% of MAPE were statistically different. Nonetheless, in the case of HDPE, the tensile strength of the composite at 8 wt% of MAPE was 12% higher than the 4 wt% of MAPE, and Young’s modulus was 4.4% lower. In the case of BioPE, and for the same MAPE contents, tensile strength was 18.5% higher and Young’s modulus 5% higher.

Strain at break increased with MAPE content. This is a consequence of the values of tensile strengths and Young’s moduli of the composites. Young’s moduli remained almost the same, thus, the slope of the stress–strain curves of the composites was similar. At the same time, composites with higher tensile strength showed longer segments for the stress–strain curve, and consequently higher strain at break. This shows the interest in researching the effects of coupling agent content on the mechanical properties of the composites. Adding reinforcement to a properly coupled composite delivers a material with enhanced strength and stiffness but noticeably decreases its ability to deform without breaking. Table 3 shows that tensile strength and stress at break of the HDPE-based composite with 8 and 4 wt% MAPE were 12.8% and 34.4% higher than uncoupled composites. BioPE-based composites returned higher strains at break than HDPE-based ones, enabling a higher contribution of the matrix to the tensile strength of the composite.

The behavior of tensile strength and Young’s modulus of the HDPE and BioPE-based composites was similar (Figure 2). Moreover, the tensile strengths of the composites with 8 wt% of MAPE showed no statistically significant differences (Table 3). On the other hand, Young’s moduli and strains at break showed such differences, and BioPE-based composites delivered the highest values. BioPE-based composites showed strains at break of 42.9% higher than HDPE-based composites. While the composition of the BioPE composite was not provided, authors think that may include a plasticiser.

Based on the results, BioPE-based composites showed competition in front of HDPE-based materials in terms of tensile properties. Thus, it is possible to find a bio-based alternative to partially bio-based materials. Another question is finding the competitiveness of the material compared with glass fiber-reinforced polyolefin. HDPE-based composites reinforced with 20 and 30 wt% of glass fiber showed tensile strengths of 37.86 and 45.19 MPa, and Young’s moduli of 4.6 and 6 GPa, respectively [62]. BioPE-based composite materials with 30 wt% AF exhibited lower tensile properties than glass fiber reinforced materials. Nonetheless, if the evolution of the tensile strength and Young’s modulus is linear, as predicted by modified rules of mixtures (Equations (1) and (2)), 40 and 50 wt% AF contents can reach tensile strengths and Young’s moduli higher than those exhibited by glass-fiber-reinforced composites. Thus, BioPe-based composites can theoretically be competitive against non-renewable-based composites.

### 4.4. Contribution of the Reinforcements to the Tensile Properties of the Composites

**Figure 3:**
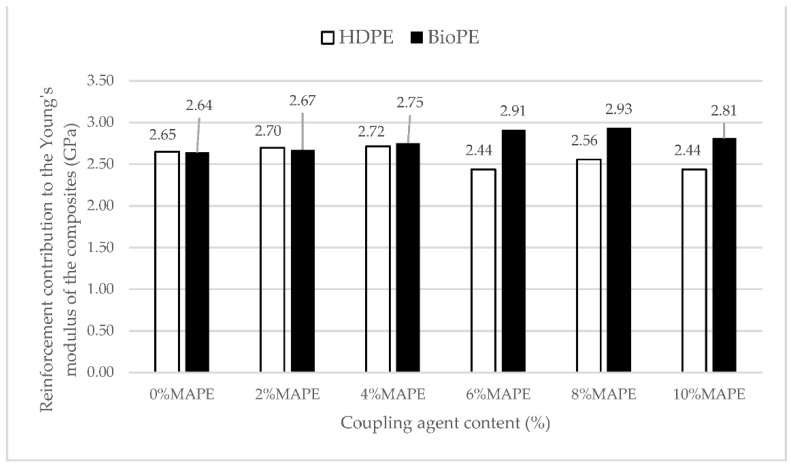
Abaca fiber contribution to Young’s moduli of HDPE and BioPE-based composites against MAPE content.

Once the competitiveness of BioPE-based materials, in terms of tensile properties, is established, it is of interest to know if the obtained properties can be enhanced. The properties of a composite depend on the contributions of the phases to such properties, and the ability to contribute is impacted by the compatibility between the phases, the morphology of the reinforcements, its dispersion, and its mean orientation. As was commented, the linear evolution of Young’s modulus is a hint of a good reinforcement dispersion in the composite material [63]. Thus, the contribution of the reinforcement to Young’s modulus of the composite can be computed by using Equation (3). Figure 3 shows the results obtained for composites reinforced with a 30 wt% of AF and MAPE contents ranging from 0 to 10 wt%.

As expected, from the data in Table 3, and due to little variation in Young’s moduli of the composites, the contributions of AF fibers remained very regular. The contribution of the matrix is expected to be $\left(1-V^{F}\right)E_{t}^{F}$, according to Equation (1), and thus constant, being 0.88 and 0.83 for the HDPE and BioPE, respectively. Thus, the variations in the contributions of the fibers follow the variations of Young’s modulus of the composite. The variations can be due to the variations in the properties of AF. Being a natural fiber, a wide scatter of its properties is expected. Nonetheless, more research is needed to discard self-entanglements of MAPE, porosity in the interface, or other causes.

**Figure 4:**
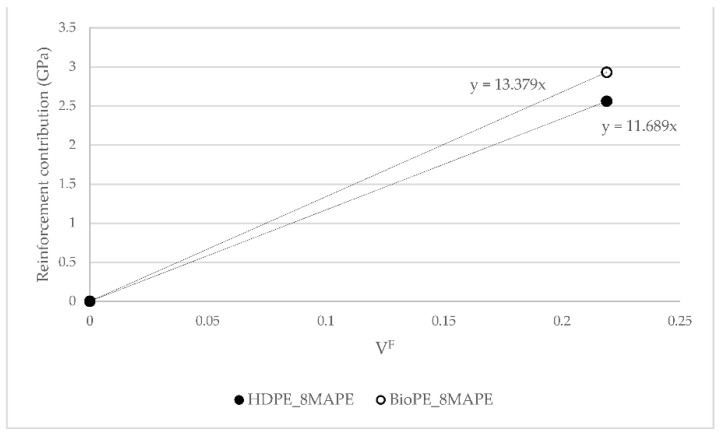
Fiber Tensile Modulus Factor (FTMF) for HDPE and BioPE AF reinforced composites.

Figure 4 shows the contribution of AF to Young’s moduli of HDPE and BioPE coupled composites.

Figure 4 was used to obtain the FTMF of AF for HDPE and BioPE. The figure shows a linear contribution of AF to Young’s moduli of the composites. The FTMF is obtained as the slope of the regression line of the contributions of the reinforcements against their content. The higher the slope of the line, the higher the contribution of the fibers and, thus, the higher the stiffening abilities of such reinforcement. FTMFs of 11.68 and 13.40 were obtained for HDPE and BioPE, respectively. The literature shows that glass fiber as PP reinforcement has a 30.13 FTMF, and thus a stiffening potential more than double AF’s [45]. Therefore, it will be necessary to more than double the content of AF to obtain composites with the same Young’s moduli as glass fiber reinforced ones. Abaca fibers as PP reinforcement showed an FTMF of 14.53, in line with the value obtained for BioPE. Other natural fibers such as spruce and barley as PP reinforcement returned 10.41 and 7.81 FTMF, respectively. Thus, abaca fibers show higher stiffening potential.

**Figure 5:**
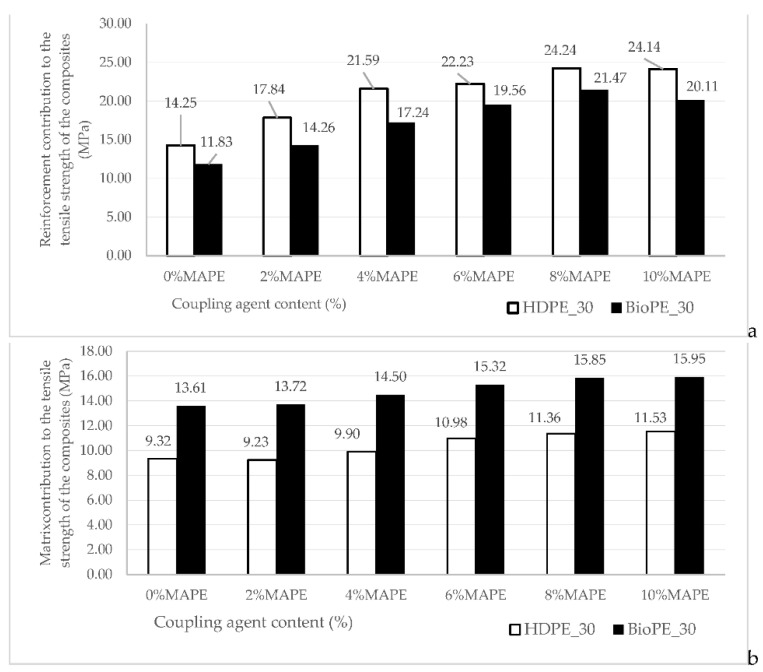
Contribution of the phases to the tensile strength of HDPE and BioPE-based composites, reinforced with 30 wt% of AF against MAPE contents; (**a**) contribution of the reinforcement; (**b**) contribution of the matrix.

As has been mentioned above, the aspects impacting such contribution are the mean orientation of the fibers, the length and length distribution of the fibers, and the intrinsic Young’s modulus of the reinforcements. A micromechanics analysis is needed to evaluate whether or not these micromechanics parameters show low variations. Before such analysis, the contribution of AF to the tensile strengths of the composites was explored. Figure 5 shows the contribution of AF to the tensile strength of HDPE and BioPE-based materials, adding different amounts of coupling agent.

**Figure 6:**
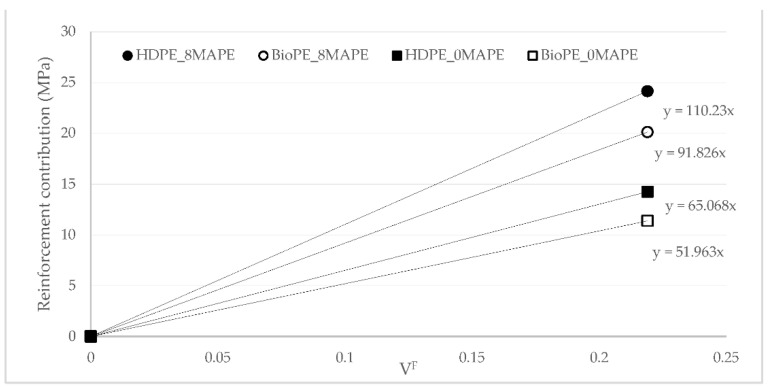
Fiber Tensile Strength Factor (FTSF) for coupled and uncoupled HDPE and BioPE AF reinforced composites.

The reinforcement contribution of AF to HDPE-based composites was found to be higher than the contribution to BioPE-based materials (Figure 5a). This can be due to the highest strain at break showed by BioPE-based composites, enabling a higher contribution of the matrix (Figure 5b). The contributions are obtained from Equation (2), and the contribution of the matrix is directly defined by the strain at the break of the composite. Thus, HDPE-based composites make better use of the strengthening potential of AF. Table 3 shows that there are no statistical differences between the values for HDPE and BioPE-based composites. While the contributions to Young’s modulus remained similar despite the percentage of MAPE (Figure 3), the contributions to the tensile strength changed noticeably with the amount of MAPE. This is an indication of the role of the coupling agent as an interface enabler. The fiber tensile strength factor (FTSF), defined by Equation (4), explores the potential of the fibers as reinforcement for a polymer. Figure 6 shows the obtained values.

FTSF shows a clear difference between coupled and uncoupled materials. As long as the intrinsic tensile strength of the reinforcement is the same, the differences have to be attributed to the coupling factor and, more specifically, to the strength of the interface. Furthermore, AF showed a higher strengthening potential for HDPE-based materials than for BioPE-based ones. Glass fiber as PP reinforcement returned a 249.51 FTSF, showing the superior strengthening abilities of this reinforcement. Hence, the amount of AF has to be 2.2 or 2.7 times higher than glass fiber to obtain composite materials with similar tensile strengths. Other natural fibers, such as cotton, delivered 122.96 and 86.99 FTSF for coupled and uncoupled PP-based materials [64]. Cotton is a fiber with high cellulose content that allows for obtaining strong interfaces, thus high FTSF is expected for coupled composites. Other fibers, such as orange tree pruning, showed 98.07 FTSF in the case of coupled composites with strong interfaces [65,66]. Thus, the FTSF shown by AF is in line with those found for other natural reinforcements, as an indication of the presence of strong interfaces for the coupled composites. A micromechanics analysis was used to characterise the interface.

### 4.5. Micromechanics Analysis

As mentioned in the presentation of the micromechanics models, the modified rule of mixtures for Young’s modulus has two unknowns that cannot be obtained directly from the tensile tests of the composites, the intrinsic Young’s modulus of the fiber (*E_t_^F^*) and the modulus efficiency factor (*η_e_*). The authors used the Hirsh equation (Equation (5)) to obtain the intrinsic Young’s moduli of the fibers. Table 4 shows the obtained values.

The values show that the intrinsic Young’s modulus of the reinforcements changes slightly with the coupling agent dosage and noticeably between the matrices. The mean value for the HDPE and BioPE-based composites was 25.7 and 28.24 GPa, respectively. This can be confusing because the fibers are the same but, on the one hand, the Hirsch model does not use the morphology of the fibers, and such morphology, especially the mean length, can change due to the viscosity of the matrix and the attrition phenomena during mixing operations. On the other hand, the intrinsic Young’s modulus obtained using micromechanics models refers to the composite and the exploitation of the reinforcing and stiffening capabilities of the fibers. Thus, BioPE-based composites seem to better exploit these capabilities, as expected from the FTMF results (Figure 4).

Table 4 shows that, for percentages of MAPE ranging from 0 to 4 wt%, the intrinsic Young’s modulus of HDPE and BioPE composites are not statistically different. Higher percentages of MAPE returned intrinsic Young’s moduli that show differences between the matrices. HDPE-based composites decreased their exploitation of the stiffening capabilities of AF, and BioPE-based composites increased such exploitation. It is also worth noting that the composites with 8 wt% of MAPE returned intrinsic Young’s moduli statistically different from the rest of the composite materials using the same matrix. It is also noticeable that 6 and 10 wt% MAPE composites returned intrinsic Young’s moduli with statistically similar values.

The micromechanics model reveals that it is theoretically possible to increase Young’s modulus of HDPE-based composites, as they do not fully exploit the stiffening capabilities of AF. This can be due to differences in the mean orientation of the fibers or their mean length. These aspects are revealed by the value of the modulus efficiency factor (*η_e_*) that it is possible to obtain from Equation (1) once the intrinsic Young’s moduli are known. The literature shows that semi-aligned short fiber reinforced composites obtain a modulus efficiency factor of around 0.5 [45,63]. Table 4 shows the obtained results. HDPE-based materials with MAPE contents from 0 to 6 wt% returned higher modulus efficiency factors than the materials adding higher MAPE contents. This indicates a change in the orientation or the mean length of the reinforcements inside the composite, causing a decrease in the potential contribution of AF to Young’s modulus of the composites. More research is needed to reveal changes in the viscosity of the composites with MAPE contents higher than 6 wt%, which can cause fiber shortenings or dispersion difficulties.

The BioPE-based composite showed a behaviour symmetrical to HDPE-based materials. The value of the modulus efficiency factor increased with the presence of MAPE up to 8 wt% contents. While the majority modulus efficiency factor obtained for the composites is in the expected range of values [0.45, 0.5], the values for the composites adding 8 wt% of MAPE are lower than 0.5, revealing that increasing Young’s modulus of the composites is theoretically possible. Using Equation (1) with a 29.9 GPa intrinsic Young’s modulus and 0.5 modulus efficiency factor for HDPE and BioPE composites returns 4.16 and 4.10 GPa Young’s moduli, respectively.

Regarding the contribution of the fibers to the tensile strength of the composite, Equation (4) can be used to evaluate the contribution of the phases. Unlike Young’s modulus, there is no direct micromechanics model to obtain the intrinsic tensile strength of AF from the tensile test data. Thus, the intrinsic tensile strength of abaca fibers was evaluated by single fiber tests and Weibull analysis. The experiments returned a theoretical limit intrinsic tensile strength of 589 MPa. This value was used to obtain a theoretical coupling factor (*f_c_*). The obtained values are shown in Table 4.

As expected, the coupling factor increased with the amount of coupling agent. The coupling factor equalises the contribution of the reinforcement by accounting for the effect of the strength of the interface, the mean orientation of the fibers, and its mean length. The increase in the strength of the interface is reflected by the tensile strength of the composites, which increases in parallel with the coupling factor. The value of the coupling factor increases for MAPE content up to 8 wt%, obtaining 0.19 and 0.17 coupling factors. These values coincide with an 8 wt% MAPE content, evaluated as the local optimum to obtain the strongest interfaces.

The literature shows that composites with strong and optimised interfaces return a coupling factor from 0.18 to 0.2. Therefore, HDPE-based composites showed strong interfaces, and BioPE-based materials showed coupling factors with the possibility to improve. Nevertheless, these results were obtained using literature values for the intrinsic tensile strength of the reinforcements.

**Figure 7:**
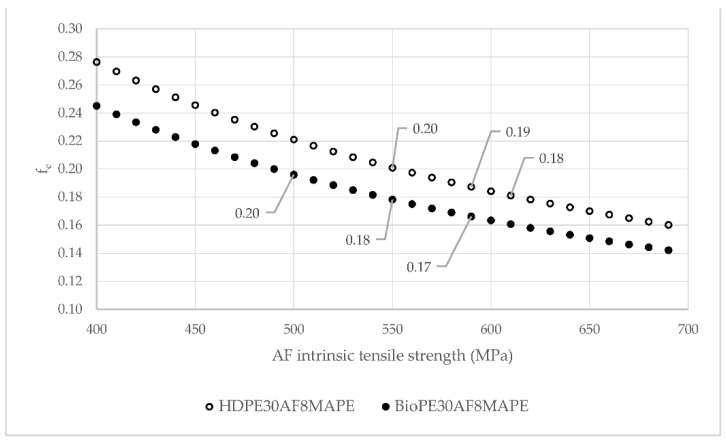
Evolution of the coupling factor of HDPE- and BioPE-based composites against Fiber Tensile Strength Factor.

Equation (4) was used to evaluate the effect of the intrinsic tensile strength of AF over the coupling factor. Figure 7 shows such evolution for HDPE and BioPE composites with 8 wt% of MAPE.

In the case of HDPE-based composites, strong interfaces with coupling factor values between 0.18 and 0.20 imply fibers with 550 to 610 MPa intrinsic tensile strengths. In the case of BioPE-based composites, the intrinsic strengths can be between 500 and 550 MPa. These strengths are inside the 430 to 1135 MPa values reported in the literature [1]. Nevertheless, BioPE-based composites returned a coupling factor below the expected for strong interfaces, indicating the possibility of improving the tensile strength of such composites. Using Equation (4) with an intrinsic tensile strength of 589 MPa, and a coupling factor of 0.2, we obtained a theoretical tensile strength of the composite of 38.17 MPa. In any case, this value has to be seen as an upper bound. More research is needed to find the causes of the decrease in the strength of the interface.

## 5. Conclusions

The determination of the tensile properties of abaca fiber-reinforced HDPE and BioPE-based composites showed that these properties were slightly impacted by the nature of the matrix. Tensile strengths obtained for composites reinforced with 30 wt% of abaca fibers were nominally higher for BioPE-based composites, but the differences were not statistically relevant at a 95% confidence rate. The strain at break of BioPE-based composites was higher than their HDPE counterparts. On the one hand, BioPE showed higher strain at break than HDPE, and on the other hand, the researchers suspect that BioPE can contain plasticisers, but this is not clear in the data provided by BioPE producers. These higher strains at break enabled higher contributions of BioPE to the tensile strength of its composites than HDPE. The contribution of the matrix is obtained as the stress–strain curve of the matrix. Thus, the higher the strain, the higher the stress and the contribution of the matrix. Tensile strength of the composites increased noticeably when a coupling agent was added. This confirms the poor adhesion between abaca fibers and the matrices, and the impact of MAPE in increasing the interactions between the phases. In the case of abaca fibers-reinforced BioPE and HDPE-based composites, the use of a coupling agent based on maleic acid showed a good strategy to increase the tensile properties. In the case of the composites studied in this paper, the highest tensile strength was obtained with 8 wt% MAPE contents.

Micromechanics analysis results obtained from modified rules of mixtures for the tensile strength and Young’s modulus showed that the interface of HDPE-based composites was stronger than their BioPE counterparts. In the case of the composites, adding 8 wt% of MAPE, HDPE, and BioPE composites returned 0.19 and 0.17 coupling factors, respectively. Having taken into account that coupling factors ranging from 0.18 to 0.20 are optimum for this kind of composite, the interface of the BioPE-based composite can be theoretically strengthened. Similarly, Young’s modulus of BioPE-based composites showed modulus efficiency factors below the expected level, denoting possible decreases in the mean length of the fibers due to attrition phenomena during mixing, having taken into account that the coupling factor is impacted by the strength of the interface, the mean length of the reinforcements, and the mean orientation of such reinforcements. Possibly, the decrease in the interface strength can be due to the same reasons as Young’s modulus.

The results showed that BioPE-based composites returned tensile properties similar to their HDPE counterparts. This can allow the use of biobased matrices instead of oil-based ones to obtain competitive composite materials.

More research is needed to evaluate the intrinsic tensile strength of AF fibers when used as HDPE and BioPE-based composites to evaluate the strength of the interface. The evaluation of the stiffness of the composites, their impact, and thermal properties, as well as their behavior when exposed to humidity, are future areas for research to fully describe and document the behavior of abaca-reinforced BioPE composites. Furthermore, the flexural and thermal properties of the materials have to be researched and documented. Finally, to establish the environmental advantages of the materials, a life cycle analysis is of interest.

## Figures and Tables

**Table 1:** Chemical composition of Abaca fibers and other natural fibers used as reinforcement.

Fiber	Cellulose	Hemicellulose	Lignin	Extractives	Ashes
Abaca	72.7	14.6	8.9	2.9	0.9
Sisal	60–78	10–22	8–14	2	-
Hemp	68–74	15–22	4–10	0.8	-
Cotton	89	4	1	0.6	-
Jute	61–71	12–20	12–13	-	-

**Table 2:** Experimental mean intrinsic tensile strengths of the abaca fibers, and the Weibull analysis outputs.

Gauge Length (mm)	Experimental σ_t_^F^ (MPa)	Weibull Shape Factor β	Characteristic Strength η (MPa)
6.35 (1/4”)	488.5 ± 112.6	5.87	530.9
12.70 (1/2”)	434.6 ± 101.9	6.01	472.5
19.05 (3/4”)	371.3 ± 107.4	6.16	409.9
25.40 (1”)	316.5 ± 108.7	6.27	356.8

**Table 3:** Experimental results for the tensile strength, Young’s modulus, and strain at break for HDPE and BioPE-based composite material reinforced with 30 wt% of AF, and the impact of coupling agent dosage.

Composite	*σ_t_^C^* (MPa)	*E_t_^C^* (GPa)	*ε_t_^C^* (%)
HDPE	18.41 ± 0.07 ^a^	1.13 ± 0.05 ^a^	8.61 ± 0.15 ^f^
HDPE30AF0MAPE	21.51 ± 0.31 ^b^	3.53 ± 0.10 ^cd^	2.26 ± 0.25 ^a^
HDPE30AF2MAPE	25.02 ± 0.54 ^c^	3.58 ± 0.04 ^fde^	2.22 ± 0.30 ^a^
HDPE30AF4MAPE	29.31 ± 0.55 ^d^	3.60 ± 0.02 ^de^	2.53 ± 0.20 ^ab^
HDPE30AF6MAPE	30.68 ± 1.35 ^e^	3.32 ± 0.05 ^b^	3.14 ± 0.75 ^bc^
HDPE30AF8MAPE	33.08 ± 0.31 ^gh^	3.44 ± 0.07 ^c^	3.40 ± 0.41 ^c^
HDPE30AF10MAPE	33.13 ± 0.38 ^gh^	3.33 ± 0.02 ^b^	3.52 ± 0.30 ^c^
BioPE	18.05 ± 0.17 ^a^	1.06 ± 0.01 ^a^	9.67 ± 0.27 ^g^
BioPE30AF0MAPE	22.46 ± 0.54 ^b^	3.47 ± 0.02 ^c^	3.18 ± 0.25 ^bc^
BioPE30AF2MAPE	24.97 ± 0.26 ^c^	3.50 ± 0.06 ^cd^	3.24 ± 0.26 ^bc^
BioPE30AF4MAPE	28.56 ± 0.70 ^d^	3.58 ± 0.02 ^de^	3.72 ± 0.27 ^cd^
BioPE30AF6MAPE	31.52 ± 0.23 ^ef^	3.74 ± 0.01 ^fg^	4.53 ± 0.30 ^de^
BioPE30AF8MAPE	33.85 ± 0.77 ^h^	3.76 ± 0.04 ^g^	4.86 ± 0.20 ^e^
BioPE30AF10MAPE	32.56 ± 0.36 ^fg^	3.64 ± 0.04 ^ef^	4.96 ± 0.21 ^e^

Different letters a, b, c, d, e, f, g and h represent the statistical difference (ANOVA, *p* < 0.05) between the properties of the materials.

**Table 4:** Influence of coupling agent contents over the tensile properties of 30 wt% AF reinforced composites.

Composite	*E_t_^F^* (GPa)	*η_e_*	*f_c_*
HDPE30AF0MAPE	26.46 ± 1.10 ^bc^	0.48 ± 0.017 ^h^	0.11 ± 0.006 ^b^
HDPE30AF2MAPE	26.99 ± 0.44 ^cd^	0.49 ± 0.006 ^cd^	0.14 ± 0.005 ^c^
HDPE30AF4MAPE	27.22 ± 0.20 ^cd^	0.49 ± 0.003 ^ab^	0.17 ± 0.005 ^f^
HDPE30AF6MAPE	24.06 ± 0.58 ^a^	0.44 ± 0.009 ^def^	0.17 ± 0.010 ^f^
HDPE30AF8MAPE	25.42 ± 0.24 ^b^	0.46 ± 0.012 ^ef^	0.19 ± 0.004 ^g^
HDPE30AF10MAPE	24.08 ± 0.07 ^a^	0.44 ± 0.004 ^a^	0.19 ± 0.004 ^g^
BioPE30AF0MAPE	26.58 ± 0.22 ^bc^	0.42 ± 0.003 ^h^	0.09 ± 0.003 ^a^
BioPE30AF2MAPE	26.95 ± 0.70 ^cd^	0.43 ± 0.010 ^fg^	0.11 ± 0.003 ^b^
BioPE30AF4MAPE	27.86 ± 0.16 ^de^	0.44 ± 0.002 ^g^	0.13 ± 0.006 ^c^
BioPE30AF6MAPE	29.63 ± 0.13 ^fe^	0.47 ± 0.001 ^ac^	0.15 ± 0.001 ^d^
BioPE30AF8MAPE	29.90 ± 0.43 ^g^	0.47 ± 0.006 ^de^	0.17 ± 0.006 ^ef^
BioPE30AF10MAPE	28.52 ± 0.47 ^ef^	0.45 ± 0.004 ^ac^	0.16 ± 0.003 ^de^

Different letters a, b, c, d, e, f, g and h represent the statistical difference (ANOVA, *p* < 0.05) between the properties of the materials.

## Data Availability

Not applicable.
